# Perceptions of Hospital Care Quality According to People Living With Multiple Long‐Term Conditions: A Scoping Review

**DOI:** 10.1111/hex.70297

**Published:** 2025-05-19

**Authors:** Freya Thompson, Sue Bellass, Thomas Scharf, Miles D. Witham, Rachel Cooper

**Affiliations:** ^1^ AGE Research Group, Translational and Clinical Research Institute, Faculty of Medical Sciences Newcastle University Newcastle upon Tyne UK; ^2^ NIHR Newcastle Biomedical Research Centre, Newcastle upon Tyne Hospitals NHS Foundation Trust, Cumbria, Northumberland, Tyne and Wear NHS Foundation Trust and Faculty of Medical Sciences Newcastle University Newcastle upon Tyne UK; ^3^ Department of Sport and Exercise Sciences Manchester Metropolitan University Manchester UK; ^4^ Population Health Sciences Institute, Faculty of Medical Sciences Newcastle University Newcastle upon Tyne UK

**Keywords:** care quality, hospital care, multimorbidity, multiple long‐term conditions, patient perceptions, scoping review, secondary care

## Abstract

**Background:**

Delivering high‐quality hospital care for people with multiple long‐term conditions (MLTC), defined as the co‐existence of two or more chronic health conditions, is important. However, evidence on care quality from the perspective of people living with MLTC has not been synthesised. The aim of this scoping review was to identify studies investigating how people living with MLTC perceive hospital care quality and to summarise key concepts and gaps in the evidence base.

**Methods:**

Systematic searches of five databases to identify all eligible studies published up until March 2024 were undertaken and supplemented by citation tracking. Peer‐reviewed articles featuring people with MLTC's perceptions of the quality of ‘usual’ care in hospitals were eligible for inclusion. All records were screened independently by two reviewers.

**Results:**

Of the 3178 titles and abstracts screened, 17 papers were eligible for inclusion (9 qualitative, 7 quantitative and 1 mixed‐methods). Studies highlighted an unmet desire for holistic interdisciplinary care (*n* = 4), prioritisation of inpatients' acute conditions over long‐term conditions (*n* = 2), barriers to patient engagement (*n* = 3) and insufficient discharge planning (*n* = 3).

**Conclusion:**

Existing studies that have investigated how people living with MLTC perceive the quality of their hospital care are diverse. However, all included studies point to ways in which hospital care for people with MLTC could be improved. The review highlights a need for studies including people of a wider range of ages, mixed‐methods studies and studies that focus on under‐researched elements of care quality, such as safety and preventative care.

**Patient or Public Contribution:**

There have been regular opportunities for engagement with the ADMISSION research collaborative's Patient Advisory Group (PAG), a group of patients and carers with lived experience of multiple long‐term conditions, who meet every 4 months. At these meetings, hospital care quality (and patients' perception thereof) has been a recurring theme, which encouraged the conceptualisation of this review. The PAG had no further direct involvement in the conduct of this review.

## 1 Introduction

Multiple long‐term conditions (MLTC), generally defined as two or more co‐occurring mental, physical or infectious health conditions lasting 12 months or more [1], are becoming more common. While prevalence estimates vary due to inconsistencies in definition and characterisation of MLTC in research [2], it is estimated that the prevalence could be between 34.9% and 39.4% in adults globally [3]. It is a concern that the prevalence of MLTC is set to rise further, especially as most health structures worldwide are not equipped to address the challenges that people living with MLTC face [4, 5]. This is because much of healthcare delivery (particularly in hospitals) and quality measurement have been designed based on the treatment of people experiencing single conditions [4, 6, 7]. Clinical guidelines, for instance, most commonly apply to people with single conditions, which has implications for the quality of care that people living with MLTC receive [8, 9].

There is growing awareness that this one‐condition management model is limited and no longer provides effective care for populations in which an increasingly high proportion of people are living with MLTC [6, 7, 10]. It is thought that a generalist and holistic approach would be more appropriate [11], yet medicine is becoming ever more specialised, both in general practice [7, 12] and in hospitals [11, 13]. Providing high‐quality care for people with MLTC in hospitals is particularly important because living with MLTC is associated with higher rates of unplanned hospital admission [14, 15, 16], longer length of stay [17], re‐admission to hospital [16] and poorer outcomes after emergency general surgery [18].

Such hospital care metrics provide important insights into the impact of MLTC on care and outcomes. Less is known, however, about how people living with MLTC perceive hospital care quality. A scoping review published in 2024 [19] synthesised qualitative evidence on experiences of hospital care from the perspectives of people living with MLTC, informal carers and healthcare professionals, finding incompatibility between the desire for patient‐centred care and the health system's shift towards clinical specialisation. Our review builds on this study by incorporating quantitative and mixed‐methods studies alongside qualitative studies with a focus specifically on people living with MLTC's perceptions of care quality. Synthesising evidence on the hospital care experiences of people living with MLTC is an important step in creating a knowledge base to inform improvements in care for this population. The aim of this review was to identify and summarise evidence of how people living with MLTC perceive the quality of care that they experience in hospitals and identify key concepts and gaps in the evidence base.

## 2 Materials and Methods

Our approach was informed by the scoping review framework devised by Arksey and O'Malley [20] and in alignment with the Preferred Reporting Items for Systematic Reviews and Meta‐Analyses extension for Scoping Reviews (PRISMA‐ScR) [21]. The review protocol, including pre‐specified inclusion and exclusion criteria, is presented in Supporting Information file 1.

### 2.1 Eligibility Criteria

The Population, Phenomenon of Interest and Context (PICo) framework was used to develop the research question and guide the search strategy (Table 1).

**Table 1:** Population, Phenomenon of interest and context (PICo) Framework.

P	Population	People living with multiple long‐term conditions (including physical, mental and infectious health conditions)
I	Phenomenon of interest	Perceptions of care quality of people living with MLTC
Co	Context	Hospitals

Eligibility criteria were as follows: peer‐reviewed qualitative, quantitative and mixed‐methods studies which investigated people with MLTC's perceptions of ‘usual’ hospital care in the context of quality of care. Some studies that also reported other stakeholders' perceptions, non‐hospital care settings and the experiences of participants with one or no long‐term conditions were included if there was ‘sufficient focus’ on hospital care quality from the perspectives of people living with MLTC. Determining whether there was ‘sufficient focus’ was guided by factors such as whether elements of hospital care quality from the perspective of people living with MLTC were explicitly mentioned in the study's aims, findings and/or conclusions, with acknowledgement that this involved a degree of subjectivity.

For the purposes of this review, hospital care was defined as inpatient or outpatient care delivered by hospital‐based clinicians. While many care quality frameworks exist [22], any study which aimed to investigate people with MLTC's perceptions of care quality was included, as long as all inclusion criteria were met.

No geographic or publication date limitations were applied, but only papers published in English were included.

### 2.2 Information Sources and Literature Search

The lead author (F.T.) and a medical librarian adapted Bellass et al.'s [19] search strategy to reflect the review objectives. This included adding the concepts of care quality and patient perceptions and removing the restriction to qualitative research. The searches involved combining both subject index and keyword terms covering the following concepts: MLTC, hospital care, care quality and patient perceptions. Full details of the search strategies can be found in Supporting Information file 2.

The electronic databases used were MEDLINE, CINAHL, ProQuest Social Sciences Premium, Scopus and Embase. The searches for all databases were executed on 20 March 2024.

Potentially eligible studies were imported into EndNote for de‐duplication, before being exported to Rayyan, where screening of titles and abstracts was carried out by three review team members (F.T., S.B. and R.C.) with two reviewers independently screening each record. Any articles where a clear inclusion or exclusion decision could not be made were discussed to achieve consensus.

Forward and backward citation tracking of included studies was conducted in May 2024.

Consistent with established scoping review methodological guidelines, we did not conduct formal critical appraisal of included studies [20], and studies were therefore not excluded on the basis of quality.

Data were extracted and synthesised from all papers that met inclusion criteria using a standard proforma by the lead author, then verified by another co‐author.

### 2.3 Synthesis and Analysis

Themes were inductively generated by the lead author. Overarching themes were presented to the review team for feedback before being narratively synthesised.

The dimension(s) of quality of care being investigated in each paper was also recorded to identify gaps in the literature and establish opportunities for further study.

## 3 Results

**Figure 1:**
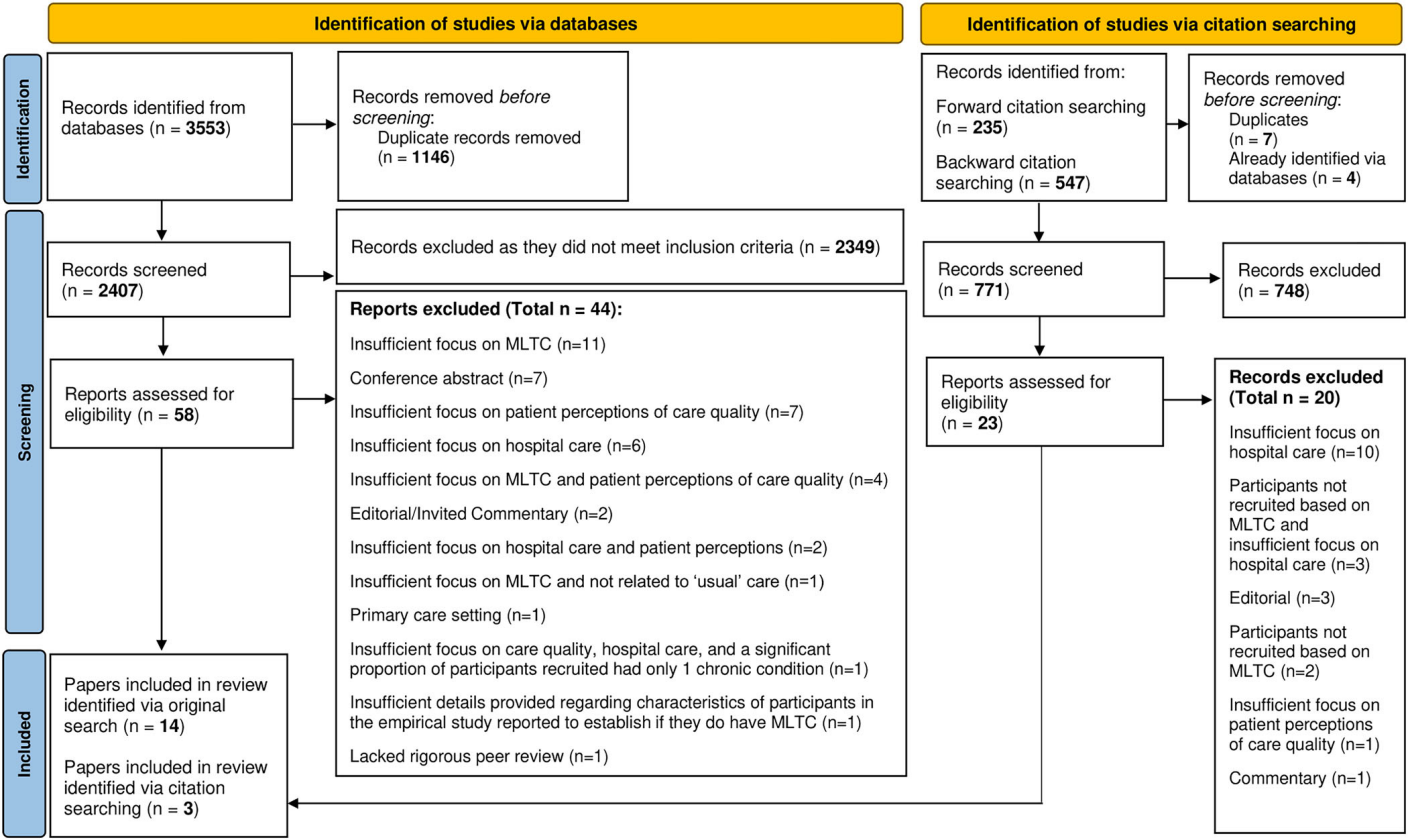
PRISMA flow diagram.

Literature searches identified 2407 unique records. After screening of titles and abstracts, 58 papers were taken forward for full‐text review, 14 of which were identified as eligible for inclusion. Citation tracking resulted in the screening of a further 771 titles and abstracts, through which a further three papers were identified. A total of 17 papers [23, 24, 25, 26, 27, 28, 29, 30, 31, 32, 33, 34, 35, 36, 37, 38, 39] were eligible for inclusion in the review (see Figure 1).

### 3.1 Characteristics of Papers

Nine of the 17 eligible papers used qualitative methods, 7 quantitative and 1 employed a mixed‐methods design. The years of publication ranged from 2004 to 2023. 5 studies were conducted in the United States, 4 in Canada, 3 in Australia and 1 each in China, England, Ethiopia, Germany and Switzerland.

Characterisations of MLTC varied (see Table 2), from 2 or more unspecified conditions [33, 36, 38], or 2 or more conditions from a predetermined list [25], to 1 specific long‐term condition with comorbidities [26, 28].

**Table 2:** Key features of included papers (ordered alphabetically by first author's surname).

First author, year and country	Characterisation of MLTC	Participant characteristics *N* Number (or %) of men; Number (or %) of women Mean age (years) Age range (years)	Aim	Methodological approach	Data collection method	Aspect of care quality
Backman et al. 2018, Canada [23]	Two or more chronic conditions	*N* = 9 (4 older adults alone, 3 family members alone, and an older adult and family member together) 6 Women; 3 men (including older adults represented by family carers) Mean age: 77.6 Age range: 56–94	To engage older adults with multiple chronic conditions and their family members in the detailed exploration of their experiences during transitions across healthcare settings and identify potential areas for future interventions.	Qualitative	Participatory visual narrative methods	Care transitions
Balbale et al. 2016, the United States [24]	Two or more chronic conditions from a predetermined list (chronic obstructive pulmonary disease, diabetes, hypertension, rheumatoid arthritis, osteoarthritis, asthma, depression, ischaemic heart disease, myocardial infarction, dementia, stroke and cancer)	*N* = 3519 95.04% men; 4.96% women Mean age: 68.1 Age range: 26–98	To use the Patient Assessment of Chronic Illness Care (PACIC) instrument to examine perceptions of chronic care among veterans with MCC.	Quantitative	Mailed survey including PACIC	Patient assessment of chronic illness care
Burgemeister et al. 2017, Switzerland [25]	People with main diagnoses of community‐acquired pneumonia, urinary tract infection, myocardial infarction, acute heart failure, deep vein thrombosis and/or pulmonary embolism, or exacerbation of chronic obstructive pulmonary disease with two or more conditions to treat	*N* = 2986 56.3% men; 43.7% women Median age: 72 Age range: not reported	To study quality measures of ED care including timeliness, patient outcome and satisfaction and identify predictors for low‐quality care.	Quantitative	Structured telephone interviews	Patient satisfaction in emergency care
Carusone et al. 2017, Canada [26]	HIV with comorbidities	*N* = 9 5 cisgender men (56%); 3 cisgender women (33%); 1 transgender woman (11%) Mean age: 42 Age range: 23–54	To investigate the obstacles and challenges faced by complex patients during hospital discharge and post‐discharge transition.	Qualitative	Longitudinal case studies	Hospital discharge plans
Eyowas et al. 2023, Ethiopia [27]	People with the simultaneous occurrence of two or more chronic non‐communicable diseases	*N* = 19 14 men (74%), 5 women (26%) Mean age: 57 Age range: 39–79	To understand the lived experiences of patients with multimorbidity and the perspective of service providers on multimorbidity and its care provision, and the capacity of the health system for managing multimorbidity in Bahir Dar City, northwest Ethiopia.	Qualitative	Semi‐structured interviews	Healthcare organisation
Hewitson et al. 2014, England [28]	Two or more long‐term conditions that are self‐reported to cause difficulties in various aspects of daily life	*N* = 65,134 (9903 of whom reported multiple limiting long‐standing conditions) 20,518 men (46.8%); 34,616 women (53.2%) Mean age: not reported Age range: 16–90+	To examine the extent to which patients with and without long‐term conditions receive self‐management support during hospital stays.	Quantitative	Survey	Interpersonal care in inpatient care
Jayakody et al. 2021, Australia [29]	People with cardiovascular disease, chronic respiratory disease, diabetes, renal disease or cancer as either a principal or secondary diagnosis when readmitted to the hospital. Readmissions primarily for mental health concerns were excluded.	*N* = 15 men; 9 women Median age: 68 Age range: 37–83	To explore the experiences and perceptions of unplanned hospital readmissions from the perspective of Aboriginal and Torres Strait Islander peoples with chronic disease.	Qualitative	Semi‐structured interviews	Unplanned readmission
Krause et al. 2006, the United States [30]	People who were enrolled in an integrated, multidisciplinary advocacy programme on the basis of health care claims over the previous 5 years	*N* = 39 15 men; 24 women Mean age: 52.39 Age range: 23–67	To examine the effects of using a multidisciplinary, integrated, whole‐person, team advocate approach to educate and empower participants with multiple chronic illnesses and coordinate, monitor and support their health care process.	Quantitative	Questionnaire pre‐ and post‐intervention	Rating of healthcare services
Kuluski et al. 2013, Canada [31]	Patients in a complex rehabilitation programme or under complex continuing care	*N* = 116 49 men (42%); 67 women (58%) Mean age: not reported Age range: below 44 to over 65	To investigate what is important in care delivery from the perspective of hospital inpatients with complex chronic disease, a currently understudied population.	Mixed‐methods	Basic statistics and semi‐structured interviews	Care delivery
Kuluski et al. 2015, Canada [32]	Multiple simultaneous chronic illnesses	*N* = 86 38 men (44.2%); 48 women (55.8%) Mean age: 61.38 Age range: 28–96	To explore factors that may serve as tipping points into poor health from the perspective of hospitalised patients with multimorbidity.	Qualitative	Structured interviews	Risk factors for health decline
Malley, Bourbonniere and Naylor 2018, the United States [33]	Two or more documented chronic conditions	*N* = 16 (11 patients and 5 family caregivers) Participants with multiple chronic conditions: 2 men; 9 women Median age: 81 Age range: Over 75	To explore how older patients with multiple chronic conditions and their family caregivers perceive their engagement and overall care experience throughout the preoperative phase of orthopaedic hip or knee joint replacement.	Qualitative	Semi‐structured telephone interviews pre‐ and post‐operatively	Patient engagement
Peltzer et al. 2020, Germany [34]	People in treatment for angiographically documented coronary heart disease and diagnosed with mental disorders	*N* = 364 (107 from hospitals, 157 from rehabilitation clinics and 100 from cardiology practices). Of these, 102 (28.0%) had mental disorders Participants with mental disorders: 60 men (58.8%); 42 women (41.2%) Mean age: not reported Age range: 35–95	To map CHD patients' (1) state of diagnostics and, if necessary, treatment of MDs, (2) trajectories and detection rate in healthcare and (3) the influence of MDs and its management on quality of life and patient satisfaction.	Quantitative	PACIC Questionnaire	Detection and treatment of mental disorders
Schenker et al. 2009, the United States [35]	Coronary heart disease and comorbidities	*N* = 703 526 men (75%); 174 women (25%) Mean age: 65 Age range: 36–97	To evaluate the association of medical comorbidities, disease severity and depressive symptoms with patient reports of doctor–patient communication in a cohort of adult patients with coronary heart disease (CHD).	Quantitative	Cross‐sectional questionnaire	Doctor–patient communication
Sun et al. 2023, China [36]	Two or more chronic diseases	*N* = 19 (10 older adults; 9 healthcare professionals) Older adults: 4 men (40%); 6 women (60%) Mean age: 74.3 Age range: 63–89	To explore barriers and facilitators in the transition of care from hospital to home for older adults in China from the perspectives of older patients with chronic diseases and healthcare professionals.	Qualitative	Semi‐structured interviews	Transitions of care from hospital to home
Wellard, Cox and Bhujoharry 2007, Australia [37]	Type 2 diabetes and cardiovascular comorbidities	*N* = 13 (7 patients; 6 nurses) Patients: 6 men; 1 woman Mean age: not reported Age range: 55–86	To identify issues that patients and cardiac specialist nurses experience with the provision of inpatient services for people undergoing cardiac surgery who also have type 2 diabetes.	Qualitative	Interviews post‐operatively and post‐discharge	Nursing care
Williams 2004, Australia [38]	Two or more chronic illnesses for approximately 5 years	*N* = 12 men (50%); 6 women (50%) Mean age: 60.91 Age range: 34–77	To explore perceptions of quality of care by patients with comorbidities who required an acute hospital stay.	Qualitative	Semi‐structured interviews	Acute care
Uga et al. 2017, the United States [39]	Comorbid chronic medical and mental illness	*N* = 116 (64 in the combined group; 52 in the separate group) 41 men; 72 women; 3 chose not to answer Mean age: not reported Age range: 70% were between 36 and 65	This study sought to compare the quality of life, satisfaction with care and utilisation of care in patients with comorbid chronic medical and mental illnesses.	Quantitative	Survey	Integrated medical and psychiatric care

Most studies (*n* = 8) recruited participants on the basis of their experience of inpatient care [26, 29, 31, 32, 33, 36, 37, 38], while four recruited from outpatient facilities [25, 27, 35, 39]. One study analysed data from an inpatient survey [28]. The remaining studies recruited from both inpatient and outpatient settings or recruited based on factors other than the type of hospital care received [23, 24, 30, 34].

While aspects of care quality investigated varied considerably, care transitions [23], including discharge [26, 36] and readmission [29], were the most common. Two studies investigated various aspects of care quality with the aim of providing a general overview of satisfaction with hospital care [24, 30]. Certain aspects of hospital care, such as nursing [37], emergency [25] and acute care [38], were also present. Two studies focused on integrated physical and mental healthcare [34, 39], while two investigated staff–patient interactions [28, 35]. The remaining studies considered healthcare organisation [27], patient engagement [29], care delivery [31] and risk factors for health decline [32].

Among the qualitative studies, the most common methodological design was cross‐sectional semi‐structured interview (*n* = 4) [27, 29, 36, 38], followed by longitudinal semi‐structured interview (*n* = 2) [33, 37], with additional instances of participatory visual narrative methods [23], longitudinal case studies [26] and cross‐sectional structured interviews [32]. The mixed‐methods study also used cross‐sectional semi‐structured interviews along with basic statistical analyses [31]. Meanwhile, the majority of quantitative studies used cross‐sectional questionnaires (*n* = 5) [24, 28, 34, 35, 39], but questionnaires pre‐ and post‐intervention [30] and cross‐sectional structured telephone interviews [25] were also utilised.

Half of the studies with a qualitative component (the qualitative studies and the mixed‐methods study) used thematic analysis (*n* = 5), and two studies used qualitative description [32, 38]. There were also instances of framework analysis [26], content analysis [33] and generic qualitative analysis [23].

All seven quantitative studies used different instruments to measure care quality, although two used either the original or a modified version of the Patient Assessment of Care for Chronic Conditions (PACIC) [24, 34]. All instruments used a scale of some form, but these differed in range from 1–4 [28, 30] to 0–100 [25]. These scales captured different elements of care quality, such as experiences of interpersonal care [28, 35], patient satisfaction [25, 30, 39] and alignment of chronic care with the chronic care model from the patients' perspective [24, 34]. The mixed‐methods paper differed in that its quantitative component investigated the socio‐demographic characteristics of hospitalised people with complex chronic disease [31], so only the qualitative data were extracted for the purposes of this review.

### 3.2 Synthesis of Findings

Five themes were identified and developed using textual narrative synthesis to summarise evidence on how people living with MLTC perceive the quality of care they experience in hospitals: ‘overall satisfaction with care’, ‘barriers to care coordination and the case for integration’, ‘patient engagement’, ‘clinician‐patient communication’ and ‘experiences of discharge’.

1. ***Overall satisfaction with care***
  A number of the included studies reported on overall satisfaction with care quality. This included one qualitative study in which Williams' [38] participants (*n* = 12) expressed their overall satisfaction with medical and nursing care in an acute setting. More of the quantitative studies reported on overall satisfaction with care, including one reporting an average Client Satisfaction Questionnaire score of 2.77 in which the maximum score was 4 [30] and another presenting an average PACIC score of 3.05 out of 5. However, these figures are difficult to interpret without comparison to groups without MLTC, which many studies did not include.
  Peltzer et al.'s [34] quantitative study compared groups of people with and without MLTC. They found that participants with both coronary heart disease and mental disorders had lower scores on 5 of 9 patient satisfaction measures when compared with people with only coronary heart disease. Hewitson et al.'s [28] results also suggested that people with MLTC report poorer satisfaction with care when compared to those without, with participants with multiple life‐limiting long‐term conditions having a higher likelihood of reporting that their care experience was negative and a lower likelihood of reporting a positive experience when compared with people with no or a single life‐limiting long‐term condition. Burgemeister's [25] study, which included some participants with only one condition, reported no correlation between the number of chronic conditions and satisfaction with care. The same lack of correlation was present among veterans with two or more long‐term conditions [24].
  There was no clear consistency across studies in characteristics associated with levels of satisfaction. For example, characteristics that were associated in univariate analyses with greater levels of satisfaction (as indicated by higher mean overall PACIC scores) included high school or less education (vs. some college or higher education), a Veteran Affairs or hospital visit in the preceding 6 months, and other race (vs. white) [24]. In contrast, Hewitson et al. [28] found that belonging to a minoritised ethnic group was associated with lower ratings of care when compared with being White British. Hewitson [28] and Burgemeister [25] also reported that shorter length of stay was associated with greater satisfaction with care, while Balbale et al. [24] found no statistically significant association. These results also refer to study populations with different characteristics, making them difficult to compare—Balbale et al.'s [24] study recruited participants with MLTC exclusively, while Burgemeister et al.'s [25] sample included some participants with only one long‐term condition and Hewitson et al.'s [28] study compared people with and without MLTC.
2. ***Barriers to care coordination and the case for integration***
  Several of the included qualitative studies presented a critical view of perceptions of care coordination and integration of care. Most of these criticisms concerned the fact that secondary care is focused on the specialist treatment of single diseases [23, 33, 38]. Two qualitative studies, for instance, reported that, rather than integrating care for all conditions experienced by the patient, acute conditions were prioritised by healthcare providers during hospital stays [33, 38], with only those conditions described as ‘highly significant to nurses', such as diabetes, attended to ([38], p. 16). This was despite the fact that Williams' [38] participants (*n* = 12) generally perceived their long‐term conditions to be more troubling than their acute illness(es). Nevertheless, the focus on acute conditions was expected by most patients [38].
  Overall, participants perceived that the lack of coordinated attention towards their long‐term conditions exacerbated their symptoms, with positive impacts on their long‐term conditions only occurring unintentionally in the midst of care for the acute illness(es) [38]. Participants were also left fearing, or indeed experiencing, new side effects, after reportedly receiving higher doses of their medications or their medications suddenly changing during their stay without explanation [29, 38].
  As people living with MLTC are required to interact with many members of staff, it was also reported that they can receive conflicting information from different healthcare professionals and be unsure who to believe [27, 38]. There was a suggestion in some studies that this may be exacerbated when patients are uninformed or unaware of which members of staff are responsible for each condition [38] and uncertain of who to contact when they need support [23]. It was also noted that poor coordination can lead to people with MLTC being sent to multiple hospitals, leading to long waits for treatment to commence or medications to be prescribed [32] and logistical issues such as frequent travel, long distances to appointments [23], parking difficulties [38] and difficulties booking appointments [38].
  Participants reported a lack of communication between care providers, whether this was between specialists [27, 38], staff on the same site [31, 38] or between GPs and the hospital [38]. Letters from specialists to GPs could be delayed or might fail to mention in‐hospital occurrences that patients had considered significant [38]. Other qualitative studies reported that participants felt that care providers did not communicate with each other [23], which may explain reports of participants having to outline their medical history to different care providers repeatedly [27], and their perceptions that improving communication between staff would optimise care [31].
  Some participants reported experiencing difficulties during a hospital stay recollecting important health information, such as the conditions they have and their impact [38]. Some used technology as aids [23, 36], while some older adults relied on family and other sources of support [33, 36]. The concept of a dedicated system coordinator to oversee care and assist in communications with other staff members was proposed by some participants [23, 31].
  Participants reported preferring consistency between care providers and care units [27, 31], expressing that long‐term relationships with care providers allow them to build trust [36]. One such example is the centralised support that local Aboriginal medical and community services have provided to some Aboriginal Australian participants [29]. Integrated services like these were found by one study to be desirable for people with MLTC, particularly highlighting a need for mental health and overall emotional support when being treated for a physical condition [31]. This is consistent with findings from Uga et al.'s [39] quantitative study, in which adults with chronic physical and mental conditions who attended an integrated clinic reported greater satisfaction with care than adults treated in separate internal medicine and psychiatry clinics. In addition, an investigation into the impact of an integrated, multidisciplinary and holistic approach to care reported a statistically significant increase in satisfaction with healthcare services (on a score from 1 [quite dissatisfied] to 4 [very satisfied]) when comparing ratings pre‐ and post‐engagement among 39 people living with MLTC [30].
3. ***Patient engagement***
  The potential importance of patient engagement was highlighted by Kuluski, Tracy and Upshur [32] in that hospitalised Canadians sometimes considered not responding to the advice of care providers a ‘tipping point’ into poor health. Several dimensions of patient engagement were found in the included studies, including informal support as an enabling factor, confidence in staff and forms of collaboration.

a. Informal support
  In some studies, engagement depended on the availability of informal support. For instance, Aboriginal Australian participants in one qualitative study reported having a strong network of family and friends who provide most of the care they need, and some felt that this had even helped avoid admissions to hospital [29]. Some hospitalised Canadian participants also felt that their health may not have worsened had they had access to more informal support. However, money was identified as a barrier to accessing this [32].
b. Adherence to care
  While many participants reported being highly engaged in their care [33], (e.g., timely medication adherence and ensuring that they attended medical appointments [29]), levels of engagement varied. Some participants, for instance, reportedly did not understand the importance of attending medical appointments [29]. Regarding adherence to medication, some expressed struggling with taking multiple medicines due to financial burden, stress and confusion [27, 29], yet described pretending to their care providers that they were taking them as prescribed [27]. Also, when participants were not informed in advance that their usual dose of medication was being altered, this led to disagreements with staff and ultimately declining to engage in care [38].
c. Passivity
  One form of patient engagement, noted by Wellard, Cox and Bhujoharry [37], was the tendency of patients with type 2 diabetes (*n* = 7) to become passive recipients of care during their hospital stay despite feeling confident and accomplished in self‐managing their condition at home. The participants interpreted this as a form of collaboration, and while many quickly returned to wanting to self‐manage, some maintained changes to their diabetes regimen post‐discharge based on healthcare professionals' recommendations [37].
d. Level of confidence in staff
  Some participants reported being unwilling to collaborate with staff, particularly nurses, with several expressing a lack of trust and confidence in health professionals' abilities [38]. In one study, patients with type 2 diabetes undergoing cardiac surgery (*n* = 7) generally perceived nurses as carers who implemented the wishes and requests of specialists, whom they perceived as being much better respected, even though most rarely acknowledged their diabetes [37]. However, nurses' help was appreciated by older adults in Backman et al.'s [23] study (*n* = 9) of care transitions.

4. ***Clinician–patient communication***

a. Factors influencing perceptions of clinicians' explanations
  During interviews in one study, people with complex chronic conditions identified better communication as one of the main aspects of care that would improve its quality [31].
  Explanations by physicians represent one key aspect of communication in hospital care. While most participants in Schenker et al.'s [35] study of doctor–patient communication, which included a sample of adults with coronary heart disease and comorbidities (*n* = 703), rated doctors' explanations of conditions as good and 27% rated communication as poor. Compared with participants who reported good explanations, those who reported receiving poor explanations were more likely to be female, Asian and less likely to be Black [35]. Ratings of communication were not associated with histories of hypertension, diabetes and myocardial infarction, nor did they differ by measures of disease severity [35]. However, each standard deviation increase in depressive symptom score in adults with coronary heart disease was associated with 50% greater odds of reporting poor explanations of conditions [35].
b. Challenges to information provision
  In other studies, some participants felt that care providers used too much medical jargon [36], leaving them feeling overloaded with information [37], which was reported to lead to distrust of medical staff [29, 36]. Conversely, some participants reported not receiving enough information, or, in the case of emergency surgery, not having enough time to absorb information [37], which may be reflected in some perceived gaps in their knowledge of the services they were receiving [29]. Participants expressed wanting to know what they can expect during their time in the hospital and at their next point of care to reduce stress and anxiety [31]. However, staff were perceived as often busy, which participants suggested could make them feel uncomfortable [31] and reluctant to ask questions [23, 27]. As a result, some reported finding information they desired elsewhere, such as from books or word of mouth from acquaintances who have gone through similar procedures [33].
  Older people with MLTC who had undergone hip or knee joint replacements and who reported lacking the digital literacy to download information booklets were sometimes surprised by unanticipated outcomes, such as particular findings during surgery, side effects from medication or mobility aids they were given upon discharge [33]. This sometimes engendered disappointment and even regret that the surgery had ever taken place [33]. It was proposed that peer support in the form of current hospital patients could have helped people with complex chronic conditions know what to expect [31].
  People living with complex chronic conditions in a Canadian study reported that sometimes the language capacities of care providers and understanding of the needs of patients from a variety of ethnic backgrounds represented a barrier to good communication [31]. Similarly, Aboriginal people in Australia expressed that information was often not explained to them by non‐Aboriginal care providers in a way they could understand [29].
c. Responding to patient preferences
  Some studies found that care providers did not instigate collaboration or encourage patients to share concerns [27, 31], which participants felt could have combatted stress [31] or health decline [32]. Neglect of patient preferences was also demonstrated in that some women experienced feeling humiliated when their preference for a same‐gender provider during bathing was not respected [31]. In another study, it was reported that people with coronary heart disease who reported poor responsiveness to patient preferences were more likely to be white and less likely to be Black than those who reported good responsiveness to patient preferences [35].
d. Perceptions of attitudes towards patients
  Tone and attitude also featured as important aspects of clinician–patient communication, as expressed by interview participants [36]. Inpatients wanted care providers to not only be good listeners, but also carry out their work enthusiastically [31]. Also important was a preference to be treated with respect and as individuals by understanding each patient's unique needs, including paying attention to non‐medical aspects of their time at the hospital [31].
  The length of in‐patient stay was perceived to affect staff's attitude towards patients, with some participants reporting that the quality of care they received decreased over time, with, for example, long response times to their call bells becoming common [38]. Quick response times were highly valued by people with MLTC, but it was reported that people found it particularly difficult to gain the attention of staff during weekends and shift changes [31]. Some inpatients also felt that certain information was not reported in nurses' handovers over time as length of stay increased, and people with MLTC even felt ‘neglected’ and ‘gossiped about’, particularly when their stay lasted more than a week [38].

5. ***Experiences of discharge***
  According to acute stay participants (*n* = 12) in Williams' study [38], discharge planning did not include care of their comorbidities. Participants felt this may have been because they were frequent consumers of health services and expected to know what to do based on prior experience [38]. Participants in Carusone et al.'s study [26], who were living with HIV and comorbidities (*n* = 9), often perceived discharge meetings to have been rushed, while older adults (*n* = 9) in Backman et al.'s study [23] felt that information had not been imparted well and that they had not felt empowered. Veterans, too, gave the lowest PACIC scores in the Follow‐Up/Coordination subscale [24]. The information recorded in the discharge plans also did not necessarily come to fruition, for example, HIV‐positive participants in Carusone's study [26] felt that the informal support received post‐discharge was not aligned with what had been previously reported, leading to isolation and loneliness.
  While some participants had positive feelings about discharge because of reasons such as returning to their families [36], the ability to re‐engage in vocational and recreational activities [31] and having more freedom [26], overall, study participants appeared to be anxious about discharge. More specifically, inpatients with complex chronic conditions were apprehensive of being ‘thrown out’ of the hospital when not ready ([31], p. 116). Older adults could fear abandonment by care providers while their symptoms persisted and reported feeling unprepared to handle any adverse reactions that might occur at home [36]. These anxieties were sometimes rooted in negative experiences of past discharge processes [36].
  Older adults understood discharge as the end of their relationship with the hospital, though they did desire continued contact [36]. Participants reported a lack of ongoing follow‐up from the hospital [36] and feeling concerned about the location, suitability and affordability of the next point of care [31]. Younger patients with complex chronic conditions were also often concerned about the impact of discharge on their spouse or partner [31]. In Sun et al.'s study [25], uncertainty about life post‐discharge led to feelings of fear, depression, loneliness and despair.

## 4 Discussion

This scoping review aimed to identify quantitative, qualitative and mixed‐methods studies that have examined how people living with MLTC perceive the quality of care they have experienced in hospitals and identify key concepts and gaps in the evidence base. Seventeen studies were identified, the findings of which were synthesised into five overarching themes.

One of the primary aims of the scoping review was to identify how hospital care quality had been characterised. Patient perceptions have been described by Donabedian [40] as ‘indispensable’, by which ‘one can obtain information about overall satisfaction and also about satisfaction with specific attributes of the interpersonal relationship, specific components of technical care and the outcomes of care’ (p. 1746). Understanding how hospital patients with MLTC perceive care quality not only offers important insights into experiences of MLTC care in secondary care settings but also facilitates reflection on the components of existing care quality frameworks.

Care coordination, for instance, has been identified for decades as an important aspect of care quality [41, 42], while continuity of care has been described previously as ‘not a unique dimension of quality but a structural characteristic that may produce benefits’ ([42], p. 1616). The issues relating to poor care coordination and integration identified in this review, such as receiving conflicting advice, poor communication between different specialisms and lack of certainty over which staff are responsible for which condition, heighten the significance of care coordination and integration as facets of overall care quality within hospital systems. That lack of communication between providers has been highlighted as a barrier to care coordination in previous reviews of MLTC studies, further emphasising the importance of this care quality component [43, 44, 45].

Care quality literature has theorised that responsibility for quality is shared between the care provider, the patient and their family—‘the practitioner may be judged blameless in some situations in which the care, as implemented by the patient, is found to be inferior’ ([40], p. 1744). The findings from this review reveal complexities around patient engagement in the context of hospital care for MLTC, with varying degrees to which patients collaborated with healthcare professionals. A recent systematic review on patient engagement interventions (goal‐setting, person‐centred care and health education) from primary care settings and hospitals for older people with MLTC [46] found significant improvements in outcomes post‐intervention in 9 of 12 included studies, suggesting that planned interventions to improve patient engagement can be successful in the MLTC context.

Interpersonal relationships between staff and patients have been described as ‘a vitally important element’ of care quality and ‘the vehicle by which technical care is implemented’ [40]. Clinician–patient communication was identified as a key theme within this review, indicating the importance people living with MLTC place on interactions with hospital staff. However, a complicated picture emerged, with, for example, some studies finding too much information had been provided, and others not enough, perhaps reflecting the diversity of different study settings. The findings from this review suggest that tailoring information to the individual patient in a culturally competent way is an important aspect of MLTC care quality. Tone and attitude in the hospital setting were also identified as important. A previous study of doctor–patient communication as perceived by people with MLTC found that family physicians score higher than hospital physicians in terms of empathic communications [47].

This review identified that it is not uncommon for people with MLTC to feel apprehensive about being discharged from the hospital. While care quality frameworks commonly include a safety component [22], this typically relates to the safety of clinical care provision rather than patient perceptions of feeling safe following treatment in a care setting. Objectively, people with MLTC are less safe than those without MLTC after discharge; previous research has demonstrated increased health utilisation and risk of mortality in the year after discharge [48], and people over 75 years old with MLTC are at greater risk of falls and loss of independence when compared to those without [49]. Further, the severity of MLTC, in terms of the number of LTC and body systems involved, has also been strongly associated with potentially avoidable readmission within 30 days [50]. Safety, as a hospital care quality component in the context of MLTC, can be conceptualised in terms of objective risks to health and clinical outcomes, but may also be understood as incorporating patient perceptions of safety.

The review also identified some topics that spanned multiple overarching themes. This included, for instance, the ways in which family and other sources of informal support can be integral to people with MLTC's hospital experience—facilitating exchanges of information, navigating care transitions and even aiding in the prevention of hospital admissions in the first place. This was also highlighted in Bellass et al.'s [19] scoping review of qualitative investigations into hospital care for people with MLTC. A common element across themes was participants' feelings of being underprepared for their hospital care, whether that might be due to a lack of provider–patient communication or inability to engage in pre‐surgical engagement. Poor care coordination also spanned themes, with logistical issues being common, transitions being perceived as rushed, participants expressing frustration at being asked the same questions repeatedly by staff, and other instances of poor communication between healthcare providers. Finally, despite several studies reporting some participants' overall satisfaction with the hospital care received, throughout all the studies, there seemed to be a desire for integrated, holistic care for people with MLTC. This is not unusual within MLTC literature, with an integrative review also identifying that people with MLTC would prefer holistic primary care [51]. In care quality literature, too, it has been emphasised that effective care is patient‐centred, taking into account the complexity of individuals, their personal experiences, wants and needs, and negotiated between the patient and their providers [42].

Several common elements of care quality frameworks were not widely considered in the included studies. This included, for example, the structure or physical environment of the hospital, such as their facilities, maintenance and other tangible aspects [22, 41, 52], safety [22, 53], preventative care [41] and equity [40, 42, 53].

This review has highlighted several potential avenues for further study (summarised in Table 3). This includes an investigation of the socio‐demographic characteristics associated with variability in perceptions of overall care quality, as required to further our understanding of health inequity. This is based on the fact that the studies included in this review were inconsistent in this regard. It is not possible to establish whether this inconsistency is attributable to the studies' different unique contexts or limitations in their design.

**Table 3:** Recommendations for further research to address gaps identified in the existing evidence base.

Observational studies	Intervention studies
Further investigation of perceptions of care quality: In different countries Across a broader range of ages Focusing on specific elements which have not yet been widely studied, including safety and equity Including comparisons of people living with and without MLTC Considering different combinations of LTC	To evaluate strategies that aim to address those factors impacting perceptions of hospital care quality for people living with MLTC identified via existing observational studies (summarised in this review)

The review also highlights a need for more studies in different countries. Over half of the studies were conducted in North America, with findings unlikely to be generalisable to other contexts because of differences in the organisation of healthcare, differences in the resources available and broader differences that would impact people with MLTC's perceptions of the quality of care that they receive.

There is currently no framework for considering quality of care that is optimised for use in studies of populations living with MLTC, which is reflected in the diverse range of approaches taken by the studies included in this review. Further study of people with MLTC's perceptions of elements of care quality, such as safety or the physical environment of hospitals, is warranted, as these were understudied aspects in existing studies.

There is also scope for more mixed‐methods studies on this topic, which would allow research questions to be examined using diverse methodological approaches, the strengths and weaknesses of each approach mutually complementing one another [54]. Also, further focus on improving care for groups with MLTC who perceive their care as the poorest, and qualitative studies utilising samples covering a broader age range may be impactful.

This review has a number of key strengths. It identifies all published, peer‐reviewed qualitative, quantitative and mixed‐methods studies that have investigated people with MLTC's perceptions of the quality of care they have received in hospitals. Several previous reviews have synthesised research on healthcare for people with MLTC [45, 55, 56, 57]. However, many of these focused exclusively on older people with MLTC, and few focused on hospital care. One recent scoping review explored experiences of MLTC hospital care from the perspectives of people with MLTC, family carers and clinicians [19], but our study differed by focusing on people living with MLTC's perceptions, concentrating on care quality and reviewing qualitative, quantitative and mixed‐methods research, as opposed to qualitative research only.

Another strength of our approach has been to identify gaps in the evidence base and determine opportunities for further novel research, which has the potential to be impactful. Further, we followed established methods for scoping reviews, including a systematic electronic search strategy, supplemented with citation tracking, to maximise the relevant peer‐reviewed studies captured.

We also must acknowledge the limitations of the review. The first concerns the omission of grey literature. This was an active decision taken a priori to ensure that the task of reviewing titles and abstracts was manageable in scale. However, this omission means that literature not published in peer‐reviewed journals will have been missed. A second limitation is that eligible studies were restricted to those written in English. As a result, insights from potentially eligible studies written in other languages have not been considered. Finally, as only 2 of the 17 papers compared the perceptions of people living with MLTC to those without MLTC, some of our findings may not be specific to people living with MLTC but instead apply to people who are hospitalised more generally.

## 5 Conclusions

A total of 17 studies were identified that investigate how people with MLTC perceive the quality of care that they experience in hospitals. The reviewed studies are diverse yet point to ways in which people with MLTC feel their hospital care could be improved. The review highlights a need for observational studies in different countries which include people of a wider range of ages and focus on elements of care quality, such as safety and equity, which have not yet been widely studied. These need to be complemented by intervention studies that assess strategies to address the factors impacting the perceptions of hospital care quality of people living with MLTC highlighted in this review.

## Author Contributions

**Freya Thompson:** conceptualisation, writing – original draft, writing – review and editing, investigation, data curation, project administration. **Sue Bellass:** conceptualisation, investigation, writing – review and editing, supervision, project administration. **Thomas Scharf:** conceptualisation, investigation, writing – review and editing, supervision. **Miles D Witham:** conceptualisation, investigation, funding acquisition, writing – review and editing, supervision. **Rachel Cooper:** conceptualisation, investigation, funding acquisition, supervision, writing – review and editing, project administration.

## Ethics Statement

This is a scoping review of published literature and so ethical approval was not required to undertake this study.

## Conflicts of Interest

The authors declare no conflicts of interest.

## Supporting information

Thompson Health Expectations Supporting Information File 1 FINAL 020525.

Thompson Health Expectations Supporting Information File 2 FINAL 020525.

## Data Availability

The authors have nothing to report.
