# Supplementary material for: Perceptions of Hospital Care Quality According to People Living With Multiple Long‐Term Conditions: A Scoping Review
Source: Health Expect. 2025 May 19;28(3):e70297. doi: 10.1111/hex.70297 (PMC12086645; doi:10.1111/hex.70297)
Supplement: Supplementary file 1 — Thompson Health Expectations Supporting Information File 1 FINAL 020525. [file HEX-28-e70297-s002.docx]

Supporting Information File 1: Study Protocol

**Title:** Perceptions of hospital care quality according to people living with multiple long-term conditions: scoping review protocol

**Review team:** Freya Thompson, Sue Bellass, Thomas Scharf, Miles D Witham and Rachel Cooper (Newcastle University)

**Aim:** The aim of this scoping review is to identify and summarise evidence of how patients living with multiple long-term conditions perceive the quality of care that they experience in hospitals, in order to identify key concepts and gaps in the evidence base. Examining the way in which care quality has been defined and assessed throughout these studies will be integral to identifying these gaps and will present opportunities for further study.

**Approach:** The scoping review framework devised by Arksey and O’Malley (1) and enhanced by Levac (2), among others, will be used to structure this review. The five core elements in the framework are as follows:

1. Identifying the research question

2. Identifying relevant studies

3. Study selection

4. Charting the data

5. Collating, summarising and reporting the results

Further detail on each stage is presented below.

1. **Identifying the research question**

*Research question framework*

The PICo framework (Population-Phenomenon of Interest-Context) has been selected for this review. Although it has been described as useful for qualitative reviews (and this review will seek quantitative and mixed methods studies in addition to qualitative literature), it has been equally associated with the analysis of social phenomena, human experience, and the perspectives of individuals (3), which aligns with the goals of this scoping review.

Within the PICo framework, ‘population’ refers to “peoples’ subjective experience or the meaning that a disease or an intervention holds for them” and the ‘phenomenon of interest’ is the “experience, event, or process under study”, the level of detail depending on the nature of the subject (3). Meanwhile, ‘context’ may refer to geographic location, demographic interest, or clinical setting (3).

**Table S1.1: Population-Phenomenon of Interest-Context (PICo) Framework**

| P | Population | People living with multiple long-term conditions (including both physical and mental health conditions) |
| --- | --- | --- |
| I | Phenomenon of Interest | Patient perceptions of care quality |
| Co | Context | Hospitals |

*Research question:* According to existing research, how have patients living with multiple long-term conditions perceived the quality of care that they receive in hospitals?

Levac *et al* recommended combining a broad research question with a “clearly articulated scope of inquiry” (2, p. 3) in order for the identification and inclusion of studies to have direction, clarity, and focus. Therefore, while the above question should be sufficiently broad enough to generate breadth of coverage without identifying an unmanageably large number of references, as recommended by Arksey & O’Malley (1), it is important to define the key concepts of ‘multiple long-term conditions’, ‘hospitals’, and ‘quality of care’.

Multiple long-term conditions (MLTC), also commonly known as multimorbidity, has been characterised in many different ways within the research literature (4). In this study, we adopt one of the most common top-level definitions of MLTC (4), that is the co-existence of two or more chronic health conditions (mental or physical) in the same individual (5).

For the purposes of this study, we will define hospital care as inpatient or outpatient care from medical specialists that does not take place at home. To elaborate, care from within traditional hospitals and community hospitals provided by secondary or tertiary care specialists will be included, while care settings such as community hospitals with primary care services, virtual wards, and other home care will be excluded.

There are many different frameworks for care quality that exist (6). Within this review, papers from any study that aims to investigate patients’ perceptions of care quality will be included, as long as it meets all other inclusion criteria (and lacks all exclusion criteria). The type(s) of quality of care being investigated in each paper will be noted and analysed along with their content. This way, the definitions of quality care being used to investigate MLTC patient perceptions of hospital care can be assessed, gaps in the literature identified, and opportunities for further study established.

1. **Identifying relevant studies**

This review will identify qualitative, quantitative, and mixed methods research studies published in peer-reviewed journals. While we acknowledge that the omission of grey literature could result in the exclusion of some important findings, after discussions with senior academic members of the review team, we opted to focus on peer-reviewed papers in order to guarantee a level of scientific rigour within the included papers. The following databases will be used: MEDLINE, CINAHL, ProQuest Social Sciences Premium, Scopus and Embase.

Scoping reviews require a sensitive and systematic searching strategy to identify and map relevant literature, ensuring that key concepts and gaps in the knowledge base can be confidently identified. However, highly sensitive strategies return considerable amounts of ineligible literature. Therefore, electronic searches will be restricted to title, abstract, and keyword fields, in order to increase the specificity of the strategy. Supplementary searches, detailed below, will be implemented to minimise the risk of overlooking potentially relevant literature.

**Table S1.2: Sample search string (MEDLINE via Ovid)**

| **Concept** | **Search string** | **Adapted from** |
| --- | --- | --- |
| Multimorbidity | 1 exp Comorbidity/  2 (co?morbidit* or multi?morbidit* or multiple LTC* or poly?morbidit* or multi?condition* or multiple chronic* or morbidity burden or multiple health problem*).ti,ab,kf.  3 ((multiple or coexisting or co-existing or concurrent or con-current or comorbid or co-morbid) adj2 (disease* or illness* or condition* or diagnos* or morbid*)).ti,ab,kf.  **4 1 or 2 or 3** | Bellass *et al*. (7) |
| Hospital | 5 (hospital* or acute setting* or inpatient* or outpatient* or ward* or secondary care or tertiary care or specialist care).ti,ab,kf.  6 exp Hospitals/  7 exp Hospitalization/  8 Inpatients/  9 Outpatients/  10 Secondary Care/  11 Tertiary Care/  **12**   **5 or 6 or 7 or 8 or 9 or 10 or 11** | Bellass *et al*. (7) |
| Care Quality | 13 “Care Quality” /  14 (quality adj3 (healthcare or care)).ti,ab,kf.  15 (standard* adj3 (healthcare or care)).ti,ab,kf.  16 (quality adj3 (outcome* or criteria)).ti,ab,kf.  **17 13 or 14 or 15 or 16** |  |
| Perceptions | 18 (satisfaction or experience* or preference* or perception* or attitude* or opinion* or view*)).ti,ab,kf.  19 Patient Satisfaction/  20 Patient Preference/  **21 18 or 19 or 20** |  |
| Combined search | **22 4 and 12 and 17 and 21** |  |
| Limiters | **23  limit 22 to English language** |  |

Line 18 in the ‘Perceptions’ concept is broad due to the potential for some studies to not use the term ‘patient’ in their titles, abstracts, or keywords. This may be especially true for circles in which person-first language is common.

*Supplementary searches*

Supplementary searches will be conducted including citation tracking, and contacting authors where appropriate.

*Documenting searches*

Records will be made of all literature searching activities, guided by the PRISMA-S checklist (8), to ensure reproducibility of the search strategy. The PRISMA-S checklist will be made available as a supplementary document.

1. **Study selection**

A team meeting was held to discuss decisions around inclusion and exclusion towards the beginning of the scoping process, as recommended by Levac (2). Consistent with scoping review methodology, studies will not be excluded on the basis of poor quality as long as they have been subject to peer-review. While systematic, scoping and narrative review studies will be excluded at the title and abstract screening stage, relevant reviews will be later catalogued in the reference management software (Endnote) to enable examination of reference lists to identify potentially eligible studies.

**Table S1.3: Eligibility criteria**

| **Inclusion criteria** | **Exclusion criteria** |
| --- | --- |
| Qualitative, quantitative, and mixed methods studies. | Study protocols, literature reviews, editorials, and commentaries. |
| Studies that report evidence of people with MLTC’s perceptions of care quality in hospitals. | Studies that report evidence of the care quality that people with MLTC experience in hospitals from the perspective of hospital staff, policy, or other viewpoints *except* patients with MLTC. That is, unless the paper includes multiple perspectives in which one is the perceptions of patients with MLTC. |
| Studies investigating hospital care – here meaning inpatient or outpatient care from medical specialists that does not take place at home. To elaborate, care from within traditional hospitals and community hospitals provided by secondary or tertiary specialists will be included. | Studies investigating hospital care at home (such as virtual wards) or in community hospitals that provide primary care services as opposed to secondary or tertiary specialists. |
| Both observational and interventional studies that include perceptions of ‘usual’ care. If a quality improvement project has measured MLTC patient perceptions of quality of care as a baseline prior to deploying an improvement intervention, this should be included. | Studies that focus purely on patient perceptions of quality improvement initiatives (with no measure of quality of care as a baseline prior to deploying the improvement). |
| Peer-reviewed papers. | Studies which have not been peer-reviewed, as well as grey literature. |
| International literature (no limit to UK-based literature, for example) |  |
| English language |  |

*Screening*

Sources will be independently screened using Rayyan, a software platform designed to support systematic reviews, by a team of researchers. Any disagreements on eligibility will be resolved in consultation with members of the supervisory team.

A PRISMA flow diagram will provide a visual representation of the study selection process, including reasons for exclusion.

1. **Charting the data**

Data will be extracted from included studies into a data charting table for ease of comparison. At a minimum the data charting table will include bibliographic information such as: author(s), year of publication, location/ setting of study, definition of multiple long-term conditions, health conditions studied, study aim and design, sample characteristics, key findings, reference to health inequalities, limitations, identified areas for future research and conceptual/ theoretical framework (if applicable). Special attention will also be given to the conceptualisations of care quality in each study, with elements such as whether the paper touches on structural care, care processes, or care outcomes (or a combination of two or more) as outlined in the work of Donabedian (9), whether a specific care quality framework has been used, or whether a specific component of care quality has been investigated, such as safety, for example. Each paper will also be given a unique identifying number (10).

1. **Collating, summarising and reporting**

The findings from the scoping review will be summarised according to key themes identified within the literature. A narrative will be created which will articulate the contours of the knowledge base, and identify gaps and areas for future research.

*Reporting*

The PRISMA-ScR (11), a reporting checklist for scoping reviews, will be completed as a supplementary document to enhance the transparency of the review process.

**References**

1. Arksey H, O'Malley L. Scoping studies: towards a methodological framework. International Journal of Social Research Methodology. 2005;8(1):19-32.

2. Levac D, Colquhoun H, O'Brien KK. Scoping studies: advancing the methodology. Implementation Science. 2010;5(69):1-9.

3. Stern C, Jordan Z, McArthur A. Developing the review question and inclusion criteria. The American Journal of Nursing. 2014;114(4):53-6.

4. Ho IS-S, Azcoaga-Lorenzo A, Akbari A, Black C, Davies J, Hodgins P, et al. Examining variation in the measurement of multimorbidity in research: a systematic review of 566 studies. 2021;6:587-97.

5. Academy of Medical Sciences. Multimorbidity: a priority for global health research. 2018.

6. Weheba G, Cure L, Toy S. Perceived dimensions of healthcare quality in published research. International Journal of Healthcare Management. 2018;13(1):357-64.

7. Bellass S, Scharf T, Errington L, Bowden Davies K, Robinson S, Runacres A, et al. Experiences of hospital care for people with multiple long-term conditions: a scoping review of qualitative research. BMC Med. 2024;22(1):25.

8. Rethlefsen ML, Kirtley S, Waffenschmidt S, Ayala AP, Moher D, Page MJ, et al. PRISMA-S: an extension to the PRISMA Statement for Reporting Literature Searches in Systematic Reviews. Syst Rev. 2021;10(1):39.

9. Donabedian A. Evaluating the Quality of Medical Care. The Milbank Memorial Fund Quarterly. 1966;44(3):166-206.

10. Daudt HML, van Mossel C, Scott SJ. Enhancing the scoping study methodology: a large inter-professional team's experience with Arksey and O'Malley's framework. BMC Medical Research Methodolgy. 2013;13(48).

11. Tricco AC, Lillie E, Zarin W, O'Brien KK, Colquhoun H, Levac D, et al. PRISMA Extension for Scoping Reviews (PRISMA-ScR): Checklist and Explanation. Annals of Internal Medicine. 2018;169(7):467-73.
