# Supplementary material for: Perceptions of Hospital Care Quality According to People Living With Multiple Long‐Term Conditions: A Scoping Review
Source: Health Expect. 2025 May 19;28(3):e70297. doi: 10.1111/hex.70297 (PMC12086645; doi:10.1111/hex.70297)
Supplement: Supplementary file 2 — Thompson Health Expectations Supporting Information File 2 FINAL 020525. [file HEX-28-e70297-s001.docx]

**Perceptions of hospital care quality according to people living with multiple long-term conditions: a scoping review**

**Supporting Information File 2: Example Search String**

**Table S2.1: Sample Search String (MEDLINE via Ovid)**

| **Concept** | **Search string** | **Adapted from** |
| --- | --- | --- |
| Multimorbidity | 1 exp Comorbidity/  2 (co?morbidit* or multi?morbidit* or multiple LTC* or poly?morbidit* or multi?condition* or multiple chronic* or morbidity burden or multiple health problem*).ti,ab,kf.  3 ((multiple or coexisting or co-existing or concurrent or con-current or comorbid or co-morbid) adj2 (disease* or illness* or condition* or diagnos* or morbid*)).ti,ab,kf.  **4 1 or 2 or 3** | Bellass, S. et al. (1) |
| Hospital | 5 (hospital* or acute setting* or inpatient* or outpatient* or ward* or secondary care or tertiary care or specialist care).ti,ab,kf.  6 exp Hospitals/  7 exp Hospitalization/  8 Inpatients/  9 Outpatients/  10 Secondary Care/  11 Tertiary Care/  **12**   **5 or 6 or 7 or 8 or 9 or 10 or 11** | Bellass, S. et al. (1) |
| Care Quality | 13 “Care Quality” /  14 (quality adj3 (healthcare or care)).ti,ab,kf.  15 (standard* adj3 (healthcare or care)).ti,ab,kf.  16 (quality adj3 (outcome* or criteria)).ti,ab,kf.  **17 13 or 14 or 15 or 16** |  |
| Patient Perceptions | 18 (satisfaction or experience* or preference* or perception* or attitude* or opinion* or view*)).ti,ab,kf.  19 Patient Satisfaction/  20 Patient Preference/  **21 18 or 19 or 20** |  |
| Combined search | **22 4 and 12 and 17 and 21** |  |
| Limiters | **23  limit 22 to English language** |  |

**References**

1. Bellass S, Scharf T, Errington L, Bowden Davies K, Robinson S, Runacres A, Ventre J, Witham MD, Sayer AA, Cooper R. Experiences of hospital care for people with multiple long-term conditions: a scoping review of qualitative research. BMC Med. 2024 Jan 17;22(1):25. doi: 10.1186/s12916-023-03220-y.
